# Alcoholic hepatitis masquerading as tumor infiltration: Reversibility after abstinence

**DOI:** 10.1002/ccr3.2448

**Published:** 2019-09-30

**Authors:** Adam B. Gluskin, Jeffrey M. Dueker, Mohamed El Hag, Kurian J. Puthenpurayil, Ramon Bataller

**Affiliations:** ^1^ Division of Gastroenterology, Hepatology, and Nutrition University of Pittsburgh Pittsburgh Pennsylvania; ^2^ Department of Pathology University of Pittsburgh Pittsburgh Pennsylvania; ^3^ Department of Radiology University of Pittsburgh Pittsburgh Pennsylvania

**Keywords:** alcoholic hepatitis, geographic fatty infiltration, hepatitis, liver biopsy

## Abstract

A subset of patients with alcoholic hepatitis present with atypical imaging resembling hepatic tumor infiltration. Our case involves a patient who was initially thought to have multiple large hepatic metastases, ultimately found to have alcoholic hepatitis. It is essential to ask about alcohol use when clinical suspicion is high.

## Graphical abstract

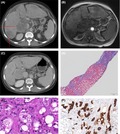

## 1 INTRODUCTON

Alcoholic hepatitis (AH) is a clinical entity often presenting as tender hepatomegaly, jaundice, fever, and anorexia in patients with prolonged alcohol abuse.^1^ An under‐appreciated consequence of AH is atypical imaging resembling massive hepatic tumor infiltration.^2^, ^3^ Whether this finding quickly reverses upon alcohol abstinence is unknown.

## 2 CASE REPORT

**Figure 1:**
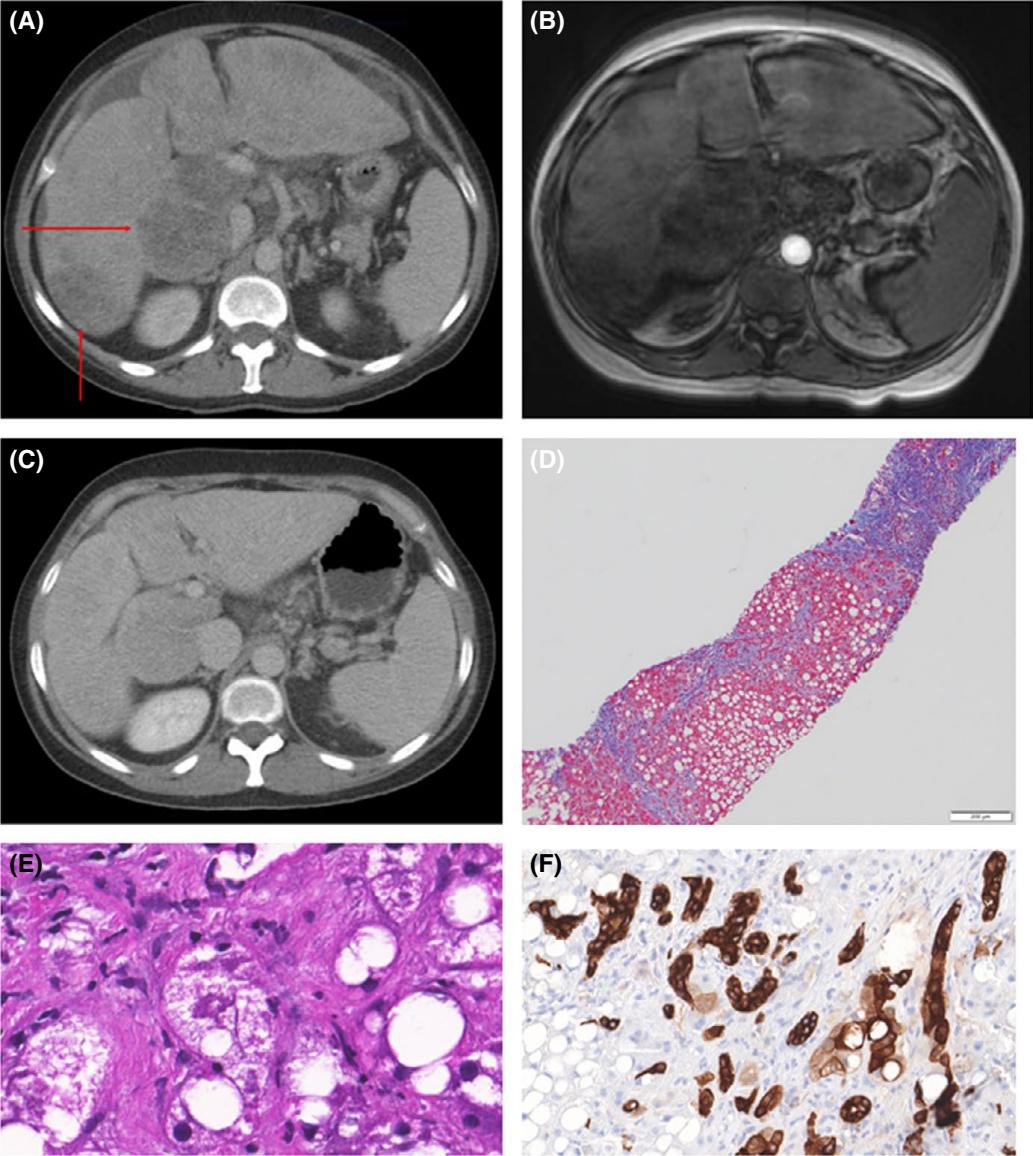
Panel A, Selected image from CT with contrast on admission shows a lobular liver with massive enlargement of the caudate and heterogeneous parenchyma. Arrows point out areas of geographic fat infiltration mimicking tumor. Note perihepatic ascites and adenopathy. Panel B, A slice through the liver on opposed phase of dual gradient echo MR shows signal loss in regions of steatosis. Panel C, Repeat CT 4 mo later with alcohol abstinence shows persistent dysmorphic appearance of the liver. Panel D, Trichrome‐stained section at 10× magnification with advanced bridging fibrosis with nodule formation, parenchymal extinction, and extensive pericellular fibrosis. Panel E, Hematoxylin and eosin staining at 100× magnification shows the liver infiltrated by mixed microvesicular‐macrovesicular steatosis with prominent ballooning degeneration, Mallory hyaline and pericellular fibrosis. Panel F, CK7 staining at 40× magnification highlights the pronounced ductular reaction

A 47‐year‐old female presented from an outside hospital because of a couple of months of increasing right upper quadrant pain as well as abdominal distention and occasional nausea and vomiting. About a month prior, she had also developed scleral icterus. Her medical history included vitamin B12 deficiency and Lyme disease. She initially reported moderate “social” alcohol use (1 drink per day). She denied a history of any illicit drug use. On admission, her vitals were unremarkable and her examination was notable for painful hepatomegaly and scleral icterus. Her initial laboratories showed an AST of 160 IU/L, ALT 36 IU/L, alkaline phosphatase 224 IU/L, total bilirubin 4.3 mg/dL, hemoglobin 9.9 gm/dL, white blood cell count 12.9 × 10^9^ cells/L, and platelet count 162  × 10^9^ cells/L. Given her symptoms, she underwent a computed tomography (CT) of the abdomen and pelvis with intravenous contrast (Figure 1A). The CT showed an enlarged, heterogeneous, and markedly dysmorphic liver, initially concerning for malignancy. There was evidence of portal hypertension including splenomegaly and moderate ascites.

At our institution, she admitted to a significant alcohol abuse history, averaging about 4 mixed drinks a day for roughly 17 years. Additional laboratories were sent revealing a GGT of 443 IU/L, CEA 5.6 ng/mL, CA19‐9 69.4 U/mL, AFP 2 ng/mL, evidence of prior immunization to hepatitis A and B, as well as negative hepatitis C and autoimmune serologies. Further imaging was obtained with a right upper quadrant ultrasound with doppler and magnetic resonance imaging (Figure 1B) which revealed a markedly cirrhotic, focally fatty infiltrated liver along with signs of portal hypertension, but with no apparent masses. In the context of all the above studies, it was felt that her symptoms were most likely secondary to AH on a background of alcoholic cirrhosis with geographic fat localization. This diagnosis was confirmed on transjugular liver biopsy (Figure 1D‐F).

The patient has remained abstinent from alcohol since discharge. She had a follow‐up CT 4 months later (Figure 1C) with a persistent cirrhotic liver morphology but with more diffuse rather than geographic fat localization suggesting that prolonged abstinence induces a redistribution of fat. Of note, she has no other obvious cause for a fatty liver.

## 3 DISCUSSION

AH is a severe clinical condition that bears a high short‐term mortality. The development of jaundice in patients with severe alcohol use disorder can be due to causes other than AH, however. Given the above, there has been a recent delineation of when to use liver biopsy to confirm the diagnosis of AH in patients with potential confounding factors like ischemic hepatitis, drug‐induced liver injury, or elevated autoimmune markers.^2^ As occurred with our patient, there seem to be certain populations that under‐report alcohol use for whom a confirmatory liver biopsy is also useful. This is especially common in women for a variety of reasons including social stigma, health insurance, and employment.

Some patients with AH develop profound and painful hepatomegaly; these patients likely have more distensible livers with more fat infiltration and even foamy degeneration as well as arterial vasodilation and ductular proliferation.^4^, ^5^ On imaging, these livers are markedly enlarged and can show a heterogenous pattern, mimicking tumoral infiltration. These patients may be less likely to have mature cirrhosis. However, they still have very elevated hepatic venous pressure gradients, are more prone to develop pseudotumoral AH, and probably have a better prognosis if they survive their index episode.

In conclusion, in patients presenting with painful hepatomegaly and jaundice and imaging suggestive of heterogeneous liver infiltration, ask about alcohol use. If there is doubt, consider a liver biopsy.

## CONFLICT OF INTEREST

None declared.

## AUTHOR CONTRIBUTIONS

ABG, JMD, MEH, KP, and RB: drafted the manuscript and involved in critical revision of the manuscript.

## INFORMED CONSENT OBTAINED FROM PATIENT

Yes.
